# A new nomogram for predicting lung metastasis in newly diagnosed endometrial carcinoma patients: A study based on SEER

**DOI:** 10.3389/fsurg.2022.855314

**Published:** 2022-07-15

**Authors:** Yufei Yuan, Ruoran Wang, Yidan Zhang, Yang Yang, Jing Zhao

**Affiliations:** ^1^ Center of Reproductive Medicine, Xi'an People's Hospital (Xi'an Fourth Hospital), Xi’an, China; ^2^ Department of Neurosurgery, West China Hospital, Sichuan University, Chengdu, China

**Keywords:** Endometrial carcinoma, lung metastases, nomogram, receiver operating characteristic curve, the surveillance, epidemiology, end results

## Abstract

**Background:**

Lung metastasis (LM) is an independent risk factor for survival in patients with endometrial cancer (EC).

**Methods:**

We reviewed data on patients diagnosed with EC between 2010 and 2015 from the Surveillance, Epidemiology, and End Results (SEER) database. The independent predictors of LM in patients with EC were identified using univariate and multivariate logistic regression analyses. A nomogram for predicting LM in patients with EC was developed, and the predictive model was evaluated using calibration and receiver operating characteristic (ROC) curves.

**Results:**

Univariate and multivariate logistic regression analyses showed that high grade; specific histological type; high tumor and node stages; larger tumor size; and liver, brain, and bone metastases were positively associated with LM risk. A new nomogram was developed by combining these factors to predict LM in patients newly diagnosed with EC. Internal and external verification of the calibration charts showed that the nomogram was well calibrated. The areas under the ROC curves for the training and validation cohorts were 0.924 and 0.913, respectively.

**Conclusion:**

We performed a retrospective analysis of 42,073 patients with EC using the SEER database, established a new nomogram for predicting LM based on eight independent risk factors, and visualized the model using a nomogram for the first time.

## Introduction

Endometrial cancer (EC) is one of the most serious epithelial malignancies that threatens women's health, ranked fourth in terms of incidence among malignancies affecting women (1). Although surgery and adjuvant radiation therapy have significantly improved disease-free and overall survival in patients with early EC (2), the prognosis in patients with metastases is poor, with a 5-year survival rate of only 17% (1, 3), respectively. EC mainly spreads *via* intra-abdominal and lymph node metastases (4), while distant organ metastasis is rare. The most common distant metastatic sites of EC are the lungs, followed by the liver, bone, and brain (5). However, compared with other gynecological malignancies, such as cervical cancer and ovarian cancer, EC has the highest frequency of lung metastasis (LM), with a 20%–25% incidence in patients with relapse (6). Although distant metastasis is a rare event in EC (7), it has a significant impact on patient survival. The survival time of patients with EC with LM is only 11 months (8). Especially for early-stage patients, doctors' insufficient judgment on the risk of distant metastasis affects the formulation of treatment and follow-up plans, resulting in decreased patient survival time. Although analysis and modeling of risk factors affecting survival and distant metastasis in EC patients have been carried out, the visualization and evaluation of predictive models for metastasis risk factors are still lacking, which leads to inconvenience in clinical application. In this study, we aimed to evaluate patients with EC registered in the SEER database from 2010 to 2015, and to develop a validated LM nomogram with high accuracy for the first time. In this way, the prognosis can be accurately made and appropriate management strategies can be selected, making it more convenient for clinical management.

## Materials and methods

### Study population

**Figure 1:**
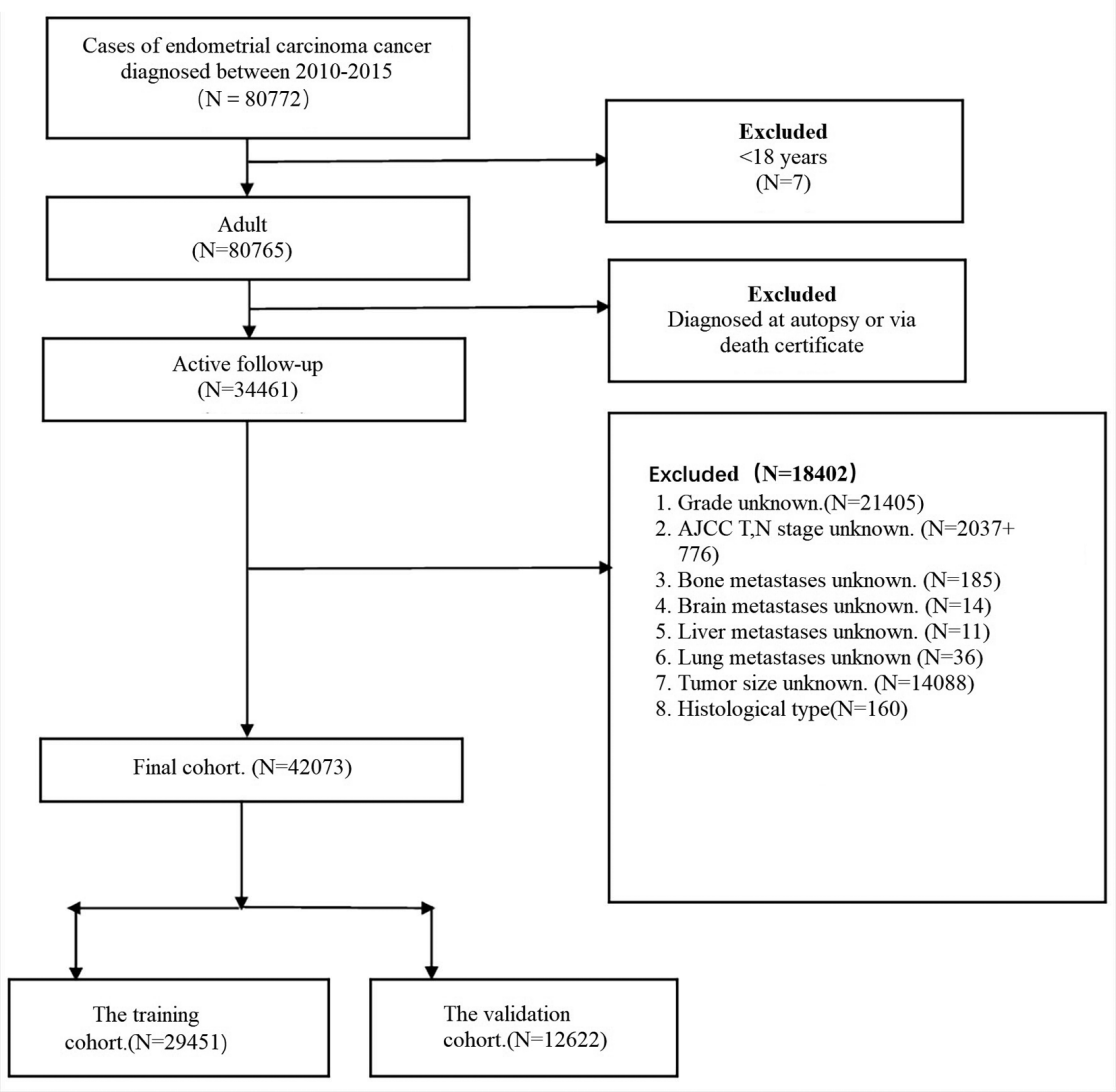
The flowchart of patient selection.

Data were obtained from the Surveillance, Epidemiology, and End Results (SEER) database. SEER *Stat 8.3.5 software (https://seer.cancer.gov/data/) was used to access the database. As details of metastases were not recorded before 2010, patients with primary EC who were ≥18 years of age at diagnosis and between 2010 and 2015 were analyzed. The site code ICD-O-3 (International Classification of Diseases Oncology-3) is limited to C54.0–C54.9 and C55.9, and the exclusion criteria for patient selection were: unknown histological type classification, unknown American Joint Committee on Cancer (AJCC) tumor (T) and node (N) staging, unknown exact tumor size, and unknown metastatic information. The patient selection flowchart is shown in Figure 1. Histological types of ECs were classified according to ICD-0-3 site/histology (endometrioid: 8380–8383/3, 8140/3, 8210/3, 8211/3, 8560/ 3, 8260/3, 8262/3, 8263/3, 8570/3, 8261/3, 8480–8482/3; serous: 8441/3, 8460/3, 8461/3; carcinosarcoma: 8950/3, 8951/3, 8980/3, 8981/3; clear cells: 8310/3 and mixed epithelial cells: 8323/3, 8255/3).

Data on clinical and pathological characteristics, including age, ethnicity, marital status at diagnosis, histological type, grade, AJCC T and N stage, tumor size, and metastatic status, were collected from the SEER database. Based on the inclusion and exclusion criteria, 42,073 EC patients were enrolled in this study. We split all populations into training and validation cohorts in a 7/3 ratio. Because all personally identifiable information in the SEER database was withheld, informed consent was not required for the use of SEER data. This research project complied with the 1964 Declaration of Helsinki and its subsequent amendments or similar ethical standards.

### Statistical analysis

Statistical analysis was performed using the SPSS 21 software. The Kolmogorov–Smirnov test was used to verify the normality of the variables. Categorical data are presented as frequency (%) and analyzed using the chi-square test. Normally distributed variables are expressed as mean ± standard deviation, whereas non-normally distributed variables are expressed as median (interquartile range). Univariate and multivariate logistic regression analyses were used to identify the risk factors. Simultaneously, 95% confidence intervals (CIs) and hazard ratios were calculated. Factors with *p* values <0.05 were included in the multivariate regression model.

The LM nomogram was also based on the results of a multivariate logistic analysis using the rms package in R version 3.4.1 (R Foundation for Statistical Computing, Vienna, Austria; www.r-project.org). A receiver operating characteristic (ROC) curve was drawn, and the area under the ROC curve (AUC) was calculated to assess the discriminative ability of the nomogram. Finally, we evaluated the stability of the LM nomogram through internal validation of 1,000 bootstrap samples. A calibration plot was drawn to analyze the agreement between the observed and predicted probabilities.

## Results

### Characteristics of the study population

Based on the selection process, 42,073 patients were included in our study. Specifically, 29,451 patients were included in the training cohort, and the remaining 12,622 patients were included in the test cohort. The incidence of LM was 1.4% (*n* = 603). The baseline of 42,073 patients are shown in Tables 1, 2. Among patients with and without LM, age (*p* = 0.002); tumor grade (*p* < 0.001), AJCC T stage (*p* < 0.001) and N stage (*p* < 0.001), and histological type (*p* < 0.001); race (*p* < 0.001); insurance status (*p* = 0.001); tumor size (*p* < 0.001); and the occurrence of bone (*p* < 0.001), brain (*p* = 0.017), and liver metastases (*p* < 0.001) were significantly different. There were no statistically significant differences in terms of the marital status (*p* = 0.236).

**Table 1:** Demographical and clinical characteristics between endometrial carcinoma patient with the lung metastases and without the lung metastases.

Variables	Total	Patients without lung metastases	Patients with lung metastases	*p*
	42,073 (100.0)	41,470 (98.6)	603 (1.4)	
Age (%)				0.014
<40	1,212 (2.9)	1,191 (2.9)	21 (3.5)	
40–49	3,650 (8.7)	3,583 (8.6)	67 (11.1)	
50–59	11,793 (28.0)	11,602 (28.0)	191 (31.7)	
60–69	14,762 (35.1)	14,577 (35.2)	185 (30.7)	
>70	10,656 (25.3)	10,517 (25.4)	139 (23.1)	
Race (%)				<0.001
White	33,660 (80.0)	33,241 (80.2)	419 (69.5)	
Black	4,018 (9.6)	3,900 (9.4)	118 (19.6)	
Other (American Indian/AK Native, Asian/Pacific Islander)	4,135 (9.8)	4,071 (9.8)	64 (10.6)	
Unknown	260 (0.6)	258 (0.6)	2 (0.3)	
Insurance status (%)				0.001
Insured	40,331 (95.9)	39,771 (95.9)	560 (92.9)	
Uninsured	1,257 (3.0)	1,226 (3.0)	31 (5.1)	
Unknown	485 (1.2)	473 (1.1)	12 (2.0)	
Marital status (%)				0.236
Married	21,472 (51.0)	21,187 (51.1)	285 (47.3)	
Unmarried	8,165 (19.4)	8,032 (19.4)	133 (22.1)	
Separated	10,472 (24.9)	10,315 (24.9)	157 (26.0)	
Unknown	1,964 (4.7)	1,936 (4.7)	28 (4.6)	
Histological type (%)				<0.001
Endometrioid histology	31,462 (74.8)	31,242 (75.3)	220 (36.5)	
Serous	3,078 (7.3)	3,026 (7.3)	52 (8.6)	
Carcinosarcoma	2,255 (5.4)	2,178 (5.3)	77 (12.8)	
Clear cell	531 (1.3)	523 (1.3)	8 (1.3)	
Mixed epithelial	2,850 (6.8)	2,813 (6.8)	37 (6.1)	
Others	1,897 (4.5)	1,688 (4.1)	209 (34.7)	
Grade (%)				<0.001
Well differentiated; Grade I	17,017 (40.4)	16,989 (41.0)	28 (4.6)	
Moderately differentiated; Grade II	11,531 (27.4)	11,462 (27.6)	69 (11.4)	
Poorly differentiated; Grade III	9,255 (22.0)	8,971 (21.6)	284 (47.1)	
Undifferentiated; anaplastic; Grade IV	4,270 (10.1)	4,048 (9.8)	222 (36.8)	
AJCC T stage (%)				<0.001
T1	33,664 (80.0)	33,482 (80.7)	182 (30.2)	
T2	3,168 (7.5)	3,069 (7.4)	99 (16.4)	
T3	4,631 (11.0)	4,373 (10.5)	258 (42.8)	
T4	610 (1.4)	546 (1.3)	64 (10.6)	
AJCC N stage (%)				<0.001
N0	37,320 (88.7)	36,949 (89.1)	371 (61.5)	
N1	2,850 (6.8)	2,710 (6.5)	140 (23.2)	
N2	1,903 (4.5)	1,811 (4.4)	92 (15.3)	
Tumor size (%)				<0.001
<2 cm	8,377 (19.9)	8,357 (20.2)	20 (3.3)	
2–5 cm	22,260 (52.9)	22,149 (53.4)	111 (18.4)	
>5 cm	11,436 (27.2)	10,964 (26.4)	472 (78.3)	
Brain metastasis (%)				<0.001
No	42,033 (99.9)	41,449 (99.9)	584 (96.8)	
Yes	40 (0.1)	21 (0.1)	19 (3.2)	
Liver metastasis (%)				<0.001
No	41,815 (99.4)	41,310 (99.6)	505 (83.7)	
Yes	258 (0.6)	160 (0.4)	98 (16.3)	
Bone metastasis (%)				<0.001
No	41,895 (99.6)	41,380 (99.8)	515 (85.4)	
Yes	178 (0.4)	90 (0.2)	88 (14.6)	

**Table 2:** Demographical and clinical characteristics between patient with the primary cohort and the validation cohort.

Variables	Total	The training cohort	The validation cohort	*p*
	42,073 (100)	29,451 (70.0)	12,622 (30.0)	
Age (%)				0.313
<40	1,212 (2.9)	874 (3.0)	338 (2.7)	
40–49	3,650 (8.7)	2,571 (8.7)	1,079 (8.5)	
50–59	11,793 (28.0)	8,257 (28.0)	3,536 (28.0)	
60–69	14,762 (35.1)	10,264 (34.9)	4,498 (35.6)	
>70	10,656 (25.3)	7,485 (25.4)	3,171 (25.1)	
Race (%)				0.186
White	33,660 (80.0)	23,565 (80.0)	10,095 (80.0)	
Black	4,018 (9.6)	2,767 (9.4)	1,251 (9.9)	
Other (American Indian/AK Native, Asian/Pacific Islander)	4,135 (9.8)	2,939 (10.0)	1,196 (9.5)	
Unknown	260 (0.6)	180 (0.6)	80 (0.6)	
Insurance status (%)				0.196
Insured	40,331 (95.9)	28,263 (96.0)	12,068 (95.6)	
Uninsured	1,257 (3.0)	863 (2.9)	394 (3.1)	
Unknown	485 (1.2)	325 (1.1)	160 (1.3)	
Marital status (%)				0.326
Married	21,472 (51.0)	15,069 (51.2)	6,403 (50.7)	
Unmarried	8,165 (19.4)	5,710 (19.4)	2,455 (19.5)	
Separated	10,472 (24.9)	7,333 (24.9)	3,139 (24.9)	
Unknown	1,964 (4.7)	1,339 (4.5)	625 (5.0)	
Histological type (%)				0.414
Endometrioid histology	31,462 (74.8)	22,063 (74.9)	9,399 (74.5)	
Serous	3,078 (7.3)	2,153 (7.3)	925 (7.3)	
Carcinosarcoma	2,255 (5.4)	1,552 (5.3)	703 (5.6)	
Clear cell	531 (1.3)	368 (1.2)	163 (1.3)	
Mixed epithelial,	2,850 (6.8)	1,963 (6.7)	887 (7.0)	
Others	1,897 (4.5)	1,352 (4.6)	545 (4.3)	
Grade (%)				0.265
Well differentiated; Grade I	17,017 (40.4)	11,865 (40.3)	5,152 (40.8)	
Moderately differentiated; Grade II	11,531 (27.4)	8,153 (27.7)	3,378 (26.8)	
Poorly differentiated; Grade III	9,255 (22.0)	6,466 (22.0)	2,789 (22.1)	
Undifferentiated; anaplastic; Grade IV	4,270 (10.1)	2,967 (10.1)	1,303 (10.3)	
AJCC T stage (%)				0.488
T1	33,664 (80.0)	23,555 (80.0)	10,109 (80.1)	
T2	3,168 (7.5)	2,252 (7.6)	916 (7.3)	
T3	4,631 (11.0)	3,223 (10.9)	1,408 (11.2)	
T4	610 (1.4)	421 (1.4)	189 (1.5)	
AJCC N stage (%)				0.082
N0	37,320 (88.7)	26,185 (88.9)	11,135 (88.2)	
N1	2,850 (6.8)	1,972 (6.7)	878 (7.0)	
N2	1,903 (4.5)	1,294 (4.4)	609 (4.8)	
Tumor size (%)				0.119
<2 cm	8,377 (19.9)	5,899 (20.0)	2,478 (19.6)	
2–5 cm	22,260 (52.9)	15,632 (53.1)	6,628 (52.5)	
>5 cm	11,436 (27.2)	7,920 (26.9)	3,516 (27.9)	
Brain metastasis (%)				0.058
No	42,033 (99.9)	29,429 (99.9)	12,604 (99.9)	
Yes	40 (0.1)	22 (0.1)	18 (0.1)	
Liver metastasis (%)				0.796
No	41,815 (99.4)	29,268 (99.4)	12,547 (99.4)	
Yes	258 (0.6)	183 (0.6)	75 (0.6)	
Bone metastasis (%)				1
No	41,895 (99.6)	29,326 (99.6)	12,569 (99.6)	
Yes	178 (0.4)	125 (0.4)	53 (0.4)	
Lung metastasis (%)				0.452
No	41,470 (98.6)	29,020 (98.5)	12,450 (98.6)	
Yes	603 (1.4)	431 (1.5)	172 (1.4)	

### Risk factors for LM in patients with EC

Among the 42,073 patients, 603 (1.4%) had LM at the first visit, and 41,470 (98.6%) did not. Univariate logistic analysis was used to analyze 11 predictors to determine LM-related variables in patients with EC. The results showed that eight predictors were related to LM in patients with EC: tumor size (*p* < 0.001), grade (*p* < 0.001), histological type (*p* < 0.001), and AJCC T stage (*p* < 0.001) and N stage (*p* < 0.001); and bone (*p* < 0.001), liver (*p* < 0.001), and brain (*p* < 0.001) metastases (Table 3). Then, statistically significant factors of the univariate logistic analysis were included in the multivariate logistic regression analysis, and the results showed that higher grade (*p *< 0.001); larger tumor (*p* < 0.001); higher T stage (*p* < 0.001); higher N stage (*p* < 0.001); specific histological type (*p* < 0.001); and the occurrence of bone (*p* < 0.001), liver (*p* < 0.001), and brain metastases (*p* < 0.001) were risk factors for LM. These predictors were evaluated for newly diagnosed patients with EC (Table 3).

**Table 3:** Univariable and multivariable logistic regression for analyzing the associated factors for developing liver metastases in endometrial carcinoma cancer patients.

Variables	Univariable			Multivariable		
	OR	95% Cl	*p* value	OR	95% Cl	*p* value
Age			0.163			
<40	References					
40–49	0.847	0.488–1.471	0.556			
50–59	0.767	0.466–1.261	0.295			
60–69	0.634	0.386–1.042	0.072			
>70	0.657	0.396–1.090	0.104			
Race			0.622			
White	References					
Black	2.530	1.983–3.228	<0.001			
Other	1.337	0.986–1.814	0.062			
Unknown	0.441	0.062–3.156	0.415			
Marital status			0.217			
Married	References			References		
Unmarried	1.259	0.987–1.607	0.064			
Separated	1.069	0.844–1.354	0.581			
Unknown	1.330	0.868–2.038	0.190			
Insurance status			0.002			0.010
Insured	References			References		
Uninsured	1.912	1.249–2.927	0.003	1.525	0.950–2.448	0.080
Others/Unknown	1.989	1.018–3.886	0.044	2.550	1.238–5.256	0.011
Tumor size			**<0**.**001**			**<0**.**001**
<2 cm	References			References		
2–5 cm	1.575	0.941–2.637	0.084	0.999	0.583–1.712	0.997
>5 cm	14.565	9.055–23.429	<0.001	2.949	1.763–4.933	<0.001
Grade			**<0**.**001**		0.000	**<0**.**001**
Well differentiated; Grade I	References			References		
Moderately differentiated; Grade II	3.340	1.999–5.582	<0.001	2.046	1.206–3.471	0.008
Poorly differentiated; Grade III	18.374	11.713–28.822	<0.001	5.749	3.509–9.419	<0.001
Undifferentiated; anaplastic; Grade IV	31.724	20.085–50.107	<0.001	5.646	3.329–9.575	<0.001
AJCC T stage			**<0**.**001**			**<0**.**001**
T1	References			References		
T2	4.993	3.691–6.755	<0.001	1.761	1.250–2.481	0.001
T3	10.746	8.587–13.448	<0.001	3.103	2.361–4.079	<0.001
T4	20.762	14.596–29.535	<0.001	4.155	2.733–6.316	<0.001
AJCC N stage			**<0**.**001**			**<0**.**001**
N0	References			References		
N1	4.967	3.916–6.302	<0.001	1.542	1.160–2.050	0.003
N2	5.384	4.101–7.069	<0.001	1.735	1.256–2.397	0.001
Bone metastasis			**<0**.**001**			**<0**.**001**
No	References			References		
Yes	79.959	55.486–115.227	<0.001	15.099	9.769–23.338	<0.001
Brain metastasis			**<0**.**001**			**<0**.**001**
No	References			References		
Yes	47.587	20.232–111.930	<0.001	10.055	3.681–27.461	<0.001
Liver metastasis			**<0**.**001**	<0.001		**<0**.**001**
No	References			References		
Yes	54.979	40.199–75.195	<0.001	8.535	5.841–12.471	<0.001
Histological type			**<0**.**001**			**<0**.**001**
Endometrioid histology	References			References		
Serous	2.460	1.722–3.513	<0.001	0.623	0.415–0.933	0.022
Carcinosarcoma	4.935	3.609–6.747	<0.001	0.914	0.639–1.308	0.624
Clear cell	0.373	0.052–2.671	<0.001	0.098	0.013–0.711	0.022
Mixed epithelial	1.981	1.322–2.968	0.326	0.863	0.557–1.336	0.508
Others	17.083	13.571–21.505	0.001	4.430	3.262–6.015	<0.001

### Development and verification of the LM diagnostic nomogram for newly diagnosed patients with EC

**Figure 2:**
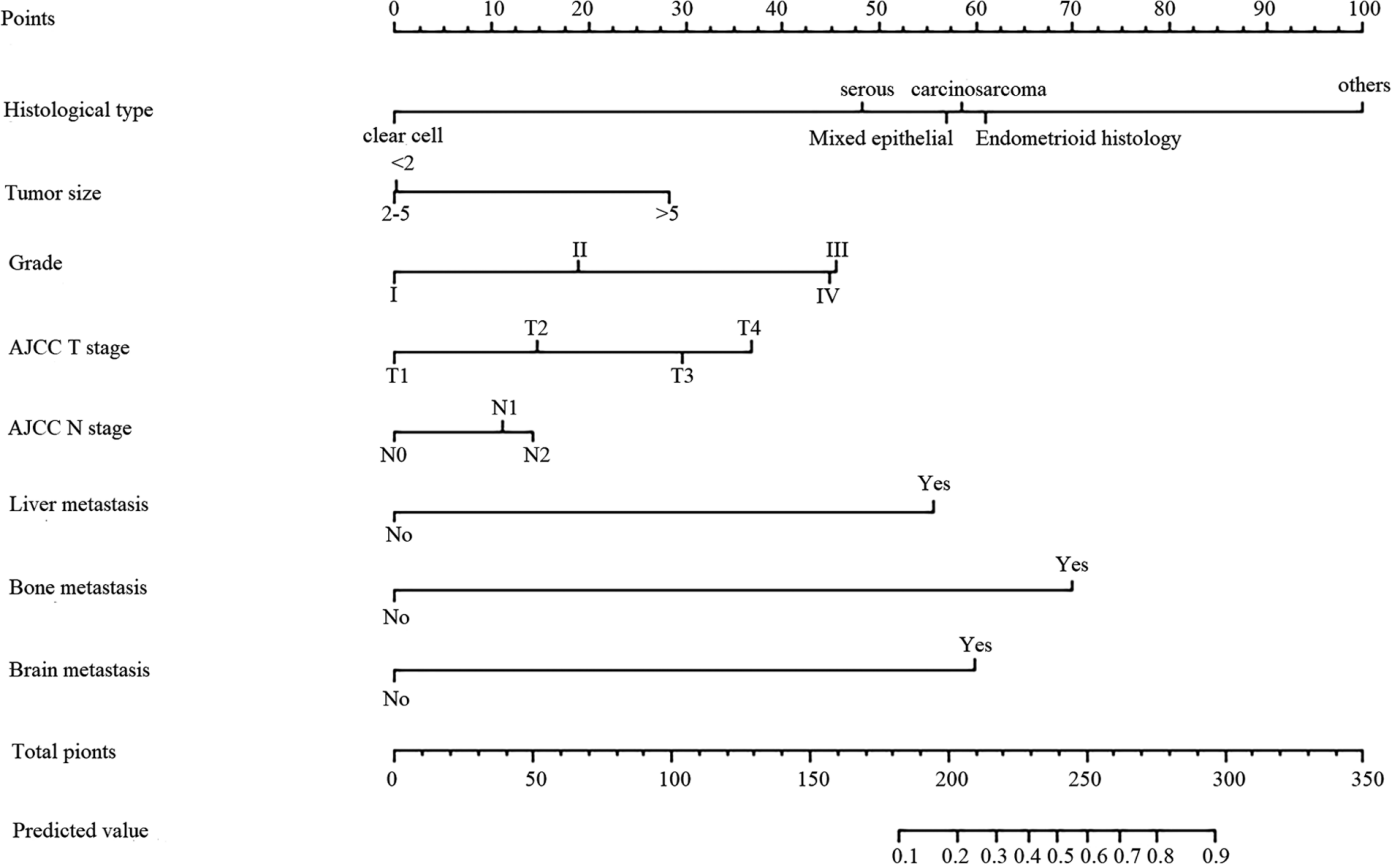
Nomogram predicting the probability of LM. The first line shows the point assignment of each variable. Lines 2–9 show the variables included in the model. When using a nomogram for a single patient, a point is assigned to each variable based on clinicopathological characteristics, and all points are added. Each score in the total score in row 9 will correspond to the probability of risk in the last row.

**Figure 3:**
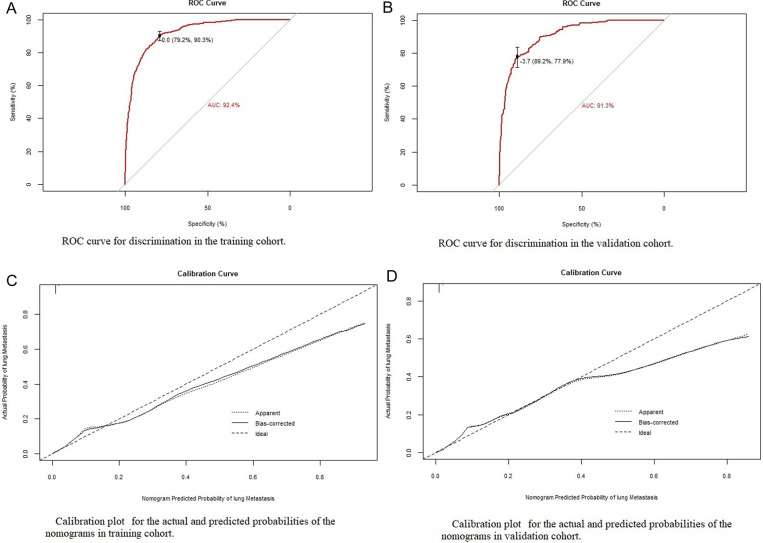
Identification and calibration of the nomogram in the training and validation cohorts. (**A**,**B**) ROC curve used to distinguish between training and validation cohorts. The area under the curve (AUC) is 0.924 and 0.913, indicating that the model has good performance. (**C**,**D**) Calibration curve of training cohort and validation cohort. The *x*-axis shows the predicted probability of the model, and the *y*-axis shows the actual probability.

Based on the eight independent LM-related variables, a diagnostic nomogram was established for LM risk assessment in newly diagnosed patients with EC (Figure 2). Simultaneously, the ROC curves of the training and validation cohorts were established. The AUC of the nomogram in the training cohort was 0.924, sensitivity was 0.903, and specificity was 0.792 (Figure 3A). The AUC of the nomogram in the validation cohort was 0.913, with a sensitivity of 0.892 and specificity of 0.779 (Figure 3B). In addition, to test the performance of the nomogram, 1,000 bootstrap resampling cycles were performed for internal verification. The calibration curves showed good agreement between the training and validation cohorts (Figures 3C,D).

## Discussion

Approximately 15%–25% of patients with EC are already at an advanced stage at the time of diagnosis. Surgery, chemotherapy, and radiotherapy are the main treatments for patients with EC (9), which can improve the survival rate of patients. Studies have found that the early detection of metastases and surgery at the primary site play an important role in improving the survival rate of patients. The median cancer-specific survival time of patients with solitary LM undergoing surgery and those not undergoing surgery is 23 months and 9 months, respectively (10). LM-directed radiotherapy can control local tumors and improve the survival rate of patients (11). Therefore, the early detection of LM from EC and appropriate clinical measures are critical for patient survival. Regarding LM in EC, there are only studies on its risk factors (12), and no predictive model has been developed to depict the risk of LM more intuitively (8) so that it can be conveniently applied in clinical management. Therefore, a clinical model for predicting LM is urgently required to guide clinical strategies and improve the survival rate of patients with LM. This study was the first to develop a nomogram using the SEER database to predict concurrent LM in patients with EC. The total score can be calculated by obtaining data on several variables on the nomogram for each patient with EC. The risk of LM can then be easily identified using the nomogram, providing guidance for further clinical management.

Our study found that the incidence of LM was 1.4%, which is similar to that in previous studies (8). We found that a high tumor grade; large tumor size; higher T and N stages; specific histological type; and presence of bone, liver (*p* < 0.001), and brain metastases (*p* < 0.001) were risk factors for LM and were included in the prediction model. Adachi et al. also found that LM is associated with stage IV disease and deep muscle infiltration (13). Jiang et al. found that large tumor size and deep muscle invasion may be risk factors for LM in patients with stage I endometrioid EC (14). Mao's clinical case study on EC found that tissue type is a risk factor for LM, and carcinosarcoma is more prone to LM (8, 13). We also found that different histological types could affect the occurrence of LM. Compared with endometrioid histology, serous and clear cells are less prone to LM, and carcinosarcoma is more prone to LM (15). In Guo's retrospective study of 730 patients, poor differentiation was an independent high-risk factor for extraperitoneal metastasis and has been widely accepted by clinicians as a feature of high-risk EC (10). Once distant metastasis occurs, it indicates that there has been extraperitoneal diffusion and cervical interstitial infiltration, therefore LM is also more likely to occur. We verified the nomogram internally and externally. There was consistency between the predicted results and those observed during verification. The ROC curve's AUC in the training cohort was 0.924 and the AUC of the line graph was 0.913 in the validation cohort. Through this predictive model, obstetricians and gynecologists will be able to estimate the likelihood of LM in patients with EC. For patients with a higher likelihood of LM, a closer follow-up should be performed. The nomogram can be used to evaluate patients with EC before chest computed tomography (CT) to determine whether the patient requires CT. If the patient's first CT evaluation cannot determine whether metastasis is present, CT follow-up should be encouraged for patients with a high risk of LM shown on the nomogram.

This study had some limitations. First, this was a retrospective study, and there are inherent biases associated with this type of study design. Additionally, only patients with LM at their first visit were analyzed. LMs occurring later in the disease were not analyzed because they may not have been recorded in the SEER database. Third, the nomograms in our study were only validated in the same population, and there may be biases in the validation of the model performance. Therefore, more clinical data should be collected for external validation of this model in the future.

## Conclusion

In conclusion, this study performed a retrospective analysis of 42,073 patients with EC using the SEER database and established for the first time a new nomogram for predicting LM based on eight independent risk factors. The verification of the model proved that it has good performance. Although predictive models have certain limitations, nomograms can reveal the relationship between clinicopathological features and LM risk in patients with EC. Through this predictive model, physicians will be able to estimate the likelihood of developing LM in patients with EC, providing guidance for further clinical management.

## Data Availability

Publicly available datasets were analyzed in this study. This data can be found here: https://seer.cancer.gov/data-software.
